# Close Association of Carbonic Anhydrase (CA2a and CA15a), Na^+^/H^+^ Exchanger (Nhe3b), and Ammonia Transporter Rhcg1 in Zebrafish Ionocytes Responsible for Na^+^ Uptake

**DOI:** 10.3389/fphys.2013.00059

**Published:** 2013-04-03

**Authors:** Yusuke Ito, Sayako Kobayashi, Nobuhiro Nakamura, Hisako Miyagi, Masahiro Esaki, Kazuyuki Hoshijima, Shigehisa Hirose

**Affiliations:** ^1^Department of Biological Sciences, Tokyo Institute of TechnologyYokohama, Japan

**Keywords:** freshwater fish, osmoregulation, mitochondria-rich cell, sodium uptake, V-ATPase, GPI anchor, proximity ligation assay, duolink

## Abstract

Freshwater (FW) fishes actively absorb salt from their environment to tolerate low salinities. We previously reported that vacuolar-type H^+^-ATPase/mitochondrion-rich cells (H-MRCs) on the skin epithelium of zebrafish larvae (*Danio rerio*) are primary sites for Na^+^ uptake. In this study, in an attempt to clarify the mechanism for the Na^+^ uptake, we performed a systematic analysis of gene expression patterns of zebrafish carbonic anhydrase (CA) isoforms and found that, of 12 CA isoforms, CA2a and CA15a are highly expressed in H-MRCs at larval stages. The *ca2a* and *ca15a* mRNA expression were salinity-dependent; they were upregulated in 0.03 mM Na^+^ water whereas *ca15a* but not *ca2a* was down-regulated in 70 mM Na^+^ water. Immunohistochemistry demonstrated cytoplasmic distribution of CA2a and apical membrane localization of CA15a. Furthermore, cell surface immunofluorescence staining revealed external surface localization of CA15a. Depletion of either CA2a or CA15a expression by Morpholino antisense oligonucleotides resulted in a significant decrease in Na^+^ accumulation in H-MRCs. An *in situ* proximity ligation assay demonstrated a very close association of CA2a, CA15a, Na^+^/H^+^ exchanger 3b (Nhe3b), and Rhcg1 ammonia transporter in H-MRC. Our findings suggest that CA2a, CA15a, and Rhcg1 play a key role in Na^+^uptake under FW conditions by forming a transport metabolon with Nhe3b.

## Introduction

Freshwater (FW) teleost fishes face a great challenge to maintain osmoregulatory homeostasis in order to survive in an extremely hypoosmotic environment ([NaCl] < 1.0 mM), which allows a constant influx of water and a diffusive loss of ions, mainly Na^+^ and Cl^−^. Therefore, FW fishes actively take up Na^+^ and Cl^−^ from their environment across the gill and skin epithelium (reviewed by Kirschner, 2004). The gill and skin contain a special type of ion-transporting epithelial cells, namely mitochondrion-rich cells (MRCs; also called ionocytes or chloride cells) which are rich in mitochondria and Na^+^–K^+^–ATPase, providing the driving force for active ion transport (Perry, 1997; Hirose et al., 2003; Evans et al., 2005; Hwang and Lee, 2007; Evans, 2008, 2010; Hwang, 2009; Lee et al., 2011; Dymowska et al., 2012; Kumai and Perry, 2012). The mechanism by which MRCs of FW fishes absorb Na^+^ has been extensively studied in traditional model species such as tilapia, trout, salmon, eel, dace, and killifish, and at least three different pathways have been proposed (for a recent review see, Hwang et al., 2011): (1) electrogenic H^+^ secretion by vacuolar-type H^+^-ATPase (H^+^-ATPase) provides driving force for Na^+^ influx through an apical amiloride-sensitive Na^+^ channel; (2) apical Na^+^/H^+^ exchanger (Nhe^1^) mediates entry of ambient Na^+^ in exchange for intracellular H^+^ equivalents; and (3) fish-specific Na^+^-Cl^−^ cotransporter (Ncc) mediates electroneutral uptake of NaCl at the apical membrane of a subpopulation of MRCs (Hiroi et al., 2008). In the latter two models, it is generally thought that the low intracellular Na^+^ concentration created by Na^+^-K^+^-ATPase is not sufficient as the driving force for electroneutral Nhe and Ncc (Parks et al., 2008).

Zebrafish, a stenohaline FW fish, is increasingly recognized as a useful model for studying the molecular basis of osmoregulatory physiology (Briggs, 2002). The availability of its genome information, together with a range of genetic approaches to manipulate gene expression, facilitates prompt identification of genes responsible for active ion transport in MRCs (Hwang, 2009). Recently, Lin et al. (2006) and Esaki et al. (2007) reported that the skin epithelium of zebrafish larvae, prior to the formation of the gills, has at least two populations of MRCs; that is, MRCs rich in Na^+^-K^+^-ATPase (NaK-MRCs) and those rich in H^+^-ATPase (H-MRCs). Of two cell types, H-MRCs profoundly secrete H^+^ from the apical plasma membrane (Lin et al., 2006) and are major sites of Na^+^ uptake (Esaki et al., 2007; Flynt et al., 2009), the activities of which largely depend on H^+^-ATPase (Esaki et al., 2007; Yan et al., 2007). In addition to H^+^-ATPase, the involvement of Nhe in Na^+^ uptake was also suggested: (1) treatments with the Nhe inhibitor ethylisopropylamiloride (EIPA) blocks Na^+^ accumulation in H-MRCs of larvae (Esaki et al., 2007) and (2) the Nhe isoform Nhe3b is expressed at the apical side of H-MRCs in the adult zebrafish gill and its expression level is dependent on environmental Na^+^ concentration and pH (Yan et al., 2007). We further showed that the carbonic anhydrase (CA) inhibitor ethoxzolamide blocked Na^+^ accumulation in H-MRCs of zebrafish larvae (Esaki et al., 2007), suggesting a contribution of CA activity to the Na^+^-uptake mechanisms. Indeed, the mRNA expression of two CA isoforms, *ca2-like a* (abbreviated here *ca2a*) and *ca15a*, have been detected in the skin MRCs of zebrafish larvae (Lin et al., 2008). Furthermore, ammonia transporter Rhcg1 has recently been demonstrated to be expressed in H-MRCs of zebrafish and suggested to have a link with the Na^+^ uptake system (Nakada et al., 2007; Kumai and Perry, 2011; Shih et al., 2012).

In this study, we determined (i) subcellular locations of the above-mentioned key players by using specific antibodies and (ii) their interactions by a proximity ligation assay (PLA). As supposed from its sequence similarity to mammalian CA IV, zebrafish CA15a was found to be expressed on the outer surface of the plasma membrane. At the apical membrane of H-MRCs, Nhe3b seemed to be working together with the other key players (i.e., CA2a, CA15a, and Rhcg1) by forming a closely associated complex.

## Materials and Methods

### Zebrafish culture

Zebrafish were maintained as described (Westerfield, 1995). The wildtype zebrafish Tubingen long fin (TL) line was generously provided by Dr. Atsushi Kawakami (Yokohama, Japan). Unless otherwise mentioned, fertilized eggs were incubated at 28.5°C in water containing 0.006% salt [60 mg ocean salt (Rohtomarine) per 1 l of distilled water (Esaki et al., 2007)], which contained 0.7 mM Na^+^ and was used as our standard FW corresponding to “Fish Water” as described in “The Zebrafish Book” (Westerfield, 1995). The concentrations of major ions of a dilute FW medium (0.0003%; 0.03 mM Na^+^; 3 mg ocean salt per liter, pH 7.2) and a concentrated medium (0.6%; 70 mM Na^+^; 6 g ocean salt per liter, pH 6.5–6.8) were previously described (Hoshijima and Hirose, 2007). Hatched or dechorionated larvae were used for the following experiments without feeding. The animal protocols and procedures were approved by the Institutional Animal Care and Use Committee of Tokyo Institute of Technology and conform to the American Physiological Society’s “Guiding Principles in the Care and Use of Laboratory Animals” (American Physiological Society, 2002).

### RNA isolation and plasmid construction

Total RNA was extracted from whole larvae at 2 days postfertilization (dpf) as described (Nakada et al., 2007). Single-stranded cDNA was synthesized from 1 μg of total RNA using Superscript III First Strand Synthesis System (Invitrogen) with oligo(dT) primer. Complementary DNA fragments containing partial or entire coding sequences of the *ca* genes were amplified by PCR with the gene-specific primer sets listed in Table 1. The partial *ca* fragments were subcloned into pBluescript SK^−^ vector (Stratagene) and used as templates for synthesis of antisense RNA probes. To construct the bacterial expression vectors for hexahistidine-tagged CA2a (full length; His_6_–CA2a), the corresponding cDNA fragment was subcloned into the *Bam*HI/*Eco*RI site of pRSET A (Invitrogen). The human leukocyte antigen (HLA)-FLAG expression vector was described previously (Saito et al., 2010). The cDNA fragments encoding TuCA15a (Tu for Tuebingen wildtype line) and TlCA15a (Tl for Tupfel long fin wildtype line) of zebrafish were amplified with the primer sets (5′-ATCAAGCTTATGATTGCCTTCCTGATCTCC-3′ and 5′-ATCGAATTCTTACAGGCAACACAAACTGGT-3′) and were then cloned into the *Hin*dIII and *Eco*RI sites of the mammalian expression vector pcDNA3 (Invitrogen, Carlsbad, CA, USA). To obtain extracellularly FLAG-tagged CA15a constructs, two oligonucleotides (5′-GATCCGATTACAAGGATGACGATGACAAGG-3′ and 5′-GATCCCTTGTCATCGTCATCCTTGTAATCG-3′) were annealed and then inserted in *Bam*HI-digested pcDNA3-CA15a vectors, yielding TuCA15a-FLAG and TlCA15a-FLAG. The sequences of all constructs were verified by sequencing.

**Table 1:** **List of primers used for PCR amplification**.

Gene	Sequence (S, sense primer; AS, antisense primer)	Accession No.
*ca2* (=*ca2a*)	S: 5′-AAGCATAACGGCCCAGACAAATG-3′ AS: 5′-GCAGTGGTGGTGTGGTCAGAGAG-3′	NM_199215
*cah-z* (=*ca2b*)	S: 5′-GCCAGAGAGTTGGGCAGAAAGC-3′ AS: 5′-GAGAGGCAGGCAGCAAGGTTTTA-3′	NM_131110
*ca4a*	S: 5′-TGGACGAGCGATTGACACCTTT-3′	XM_677649
	AS: 5′-CCAGGGGAATGGGCTTTTCA-3′	
*ca4b*	S: 5′-CGCCTATCAACATCATCACAAGCA-3′	CD758578
	AS: 5′-CCGTCCACAAAACAGCTTCTTCAC-3′	
*ca4c* (=*ca15a*)	S: 5′-GCACTTCTGTGTGAATCCGTCCA-3′	CN509883
*ca4d*	S: 5′-GCAGCGTGTGGAAACAATAAACAGTC-3′	DT079722
	AS: 5′-GTGAGTGAGCCCTCGTAGCGAAA-3′	
*ca5*	S: 5′-ATCAGCAGGACCGAGAGCAGGT-3′	CT614270
	AS: 5′-TCGTCGTACTCCACCAGAAACGA-3′	
*ca7*	S: 5′-CATCACTGGGGATACGGAGAGGA-3′	NM_200813
	AS: 5′-TTGGCAGGAGGCACTTAGGGTTA-3′	
*ca8*	S: 5′-TACTGGAGGTCCTCTCCCCAGTG-3′	NM_001017571
	AS: 5′-ACCACCCTGTCACTCAGTGGTTG-3′	
*ca9*	S: 5′-ACCAAGATCAGGACGCATGGTTA-3′	XM_689890
	AS: 5′-AGACAAGGAGGGGTCGTCAGAGA-3′	
*ca10*	S: 5′-ACTTTGGGAGTGAGGACGGACAG-3′	NM_001037121
	AS: 5′-GAAGTTGATGTTGGTGCGAATGC-3′	
*ca14*	S: 5′-CATAGCAACAGGGAGCGAACACA-3′	NM_001037693
	AS: 5′-ATCTTGCATTCTGGGCTGAGGAG-3′	
*rpl9*	S: 5′-GTACAAAATGCGATCTGTATATGCC-3′	NM_001003861
	AS: 5′-TAATTTAGTCCTCTTGCTGTTCCAC-3′	

The reason why we prepared two CA15a constructs (TuCA15a-FLAG and TlCA15a-FLAG) is that we first used, for preparing anti-peptide antibody, the CA15a sequence in the zebrafish database, which was obtained from the Tuebingen wildtype line, but the CA15a sequence of our line (Tupfel long fin wildtype line) was later found to be different in the region (residues 21–37) used as antigen peptides for TuCA15a (NSAVAWCYNN*PL*CNFTT) and TlCA15a (NSAVAWCYNN*SS*CNFTT).

### Antibodies

To construct a hexahistidine-tagged zCA2a, a cDNA fragment encoding the whole zCA2a sequence was subcloned into the *Bam*HI/*Eco*RI sites of the bacterial expression vector pRSET (Invitrogen). The recombinant proteins were purified with Talon metal affinity resin (Clontech) as in the manufacturer’s instructions. BL21(DE3) pLysS cells (Novagen) transformed with the expression vector were used to inoculate 1 l of LB broth containing 100 μg/ml of ampicillin. Protein expression was then induced by adding isopropyl-1-thio-d-galactopyranoside to a final concentration of 1 mM for 4 h at 37°C. Then the cells were harvested from the culture by centrifugation and resuspended in 40 ml of PBS, followed by freezing-thawing and sonication disruption. After centrifugation (10,000 *g* at 4°C), supernatants were purified with Talon metal affinity resin. Following purification, recombinant proteins were dialyzed against saline at 4°C. Polyclonal antibodies were prepared in Japanese white rabbits by injecting 200 μg of purified recombinant proteins, emulsified with the adjuvant TiterMax Gold (CytRx) (1:1), intramuscularly at multiple sites. The rabbits were injected three times at 1-month intervals and whole blood was collected after the third immunization. Polyclonal antiserum specific for CA15a and Nhe3b were each made in rabbit and rat which were immunized with a keyhole limpet hemocyanin (KLH)-conjugated synthetic peptide corresponding to residues 838–851 of zebrafish Nhe3b (ETPEEKPATHHTRL) and 21–37 of TuCA15a (NSAVAWCYNNPLCNFTT) (Operon Biotechnologies, Tokyo, Japan). Rat polyclonal antiserum against the H^+^-ATPase β subunit of dace (*Tribolodon hakonensis*) Atp6v0b (Hirata et al., 2003), rat polyclonal antiserum against the α subunit of eel (*Anguilla japonica*) Na^+^/K^+^-ATPase (Mistry et al., 2001), rabbit polyclonal antiserum against dace Nhe3 (Hirata et al., 2003) and rabbit polyclonal antiserum against zRhcg1 (Nakada et al., 2007) were described previously. The specificity of the anti-zCA2 and anti-zCA15a antisera was evaluated by immunoprecipitation of ^35^S-labeled antigen (Figure A2 in Appendix) as described below. When used for immunohistochemistry, the anti-CA2a, anti-CA15a, and anti-H-ATPase antibodies gave stronger signals on the larval skin than the gill whereas the anti-Nhe3b yielded stronger signals on gill sections.

### Cell culture and transfection

Human embryonic kidney fibroblast 293T cells and COS7 cells were cultured in Dulbecco’s Modified Eagle Medium containing 10% fetal bovine serum (FBS), 100 U/ml penicillin, and 100 μg/ml streptomycin at 37°C in a humidified atmosphere containing 5% CO_2_. Transient transfection of plasmid DNA was performed using TransFectin reagent (Bio-Rad, Hercules, CA, USA) according to the manufacturer’s instruction.

### Subcellular fractionation

Two days after transfection, cells were homogenized and sonicated in ice-cold PBS containing protease inhibitors (10 mM leupeptin, 1 mM pepstatin, 5 mg/ml aprotinin, and 1 mM phenylmethylsulfonyl fluoride). The homogenates were centrifuged at 60,000 rpm in a TLA100.3 rotor (Beckman Coulter, Fullerton, CA, USA) for 20 min. The supernatant (the cytosolic fraction) was recovered, and the membrane pellet was resuspended and homogenized in lysis buffer (10 mM sodium phosphate, pH 7.4, 1% Nonidet P-40, 0.5% sodium deoxycholate, 0.1% SDS, 0.15 M NaCl, 2 mM EDTA, 50 mM NaF, 1 mM Benzamidine) containing the protease inhibitors.

### Immunoprecipitation

Cells were homogenized in 1 ml of lysis buffer (1 M urea, 50 mM Tris, pH 7.4, 150 mM NaCl, 1% Nonidet P-40, 10% glycerol, 1 mM EDTA, and 1 mM dithiothreitol) containing the protease inhibitors. After centrifugation to remove cell debris, the homogenates were incubated with 20 μl of anti-FLAG M2 affinity gel (Sigma-Aldrich, St. Louis, MO, USA) for 12 h at 10°C. After extensive washing with lysis buffer, the bound materials were eluted with acid buffer (1% Nonidet P-40 and 0.1 M glycine, pH 3.5).

### *In vitro* translation

*In vitro* translation was performed by using the TNT T7 Quick Coupled Transcription/Translation System (Promega, Madison, WI, USA) with [^35^S]methionine and [^35^S]cysteine (PerkinElmer, Boston, MA, USA) according to the manufacturer’s instruction. The labeled proteins were separated by SDS–PAGE, and were then exposed to an Imaging Plate (Fujifilm, Tokyo, Japan). The signals were analyzed by using FLA7000 (Fujifilm).

### Immunofluorescence microscopy

Cells were fixed with 4% PFA in PBS for 10 min and were then blocked with 5% FBS in PBS for 30 min at room temperature. The cells were incubated with anti-FLAG M2 antibody (Sigma, St. Louis, MO, USA) at a 1:1,000 dilution in PBS for 30 min at room temperature. After washing with PBS, the cells were incubated with Alexa Fluor 488-labeled anti-mouse IgG secondary antibodies (diluted 1:1,000; Molecular Probes, Eugene, OR, USA) for 30 min at room temperature. The signals were detected by an IX70 fluorescence microscopy (Olympus, Tokyo, Japan).

### Whole mount *in situ* hybridization

Using the linearized *ca* plasmid vectors as templates, digoxigenin (DIG)-labeled antisense RNA probes were synthesized by *in vitro* transcription with T3 and T7 RNA polymerase (Stratagene) and digoxigenin (DIG) RNA labeling Mix (Roche). Whole mount *in situ* hybridization was performed as described (Esaki et al., 2009), with the following minor modifications: the larvae were permeabilized by treating with 10 μg/ml proteinase K for 2 min after rehydration. Combination with immunohistochemistry was performed with anti-dace Atp6v0b antibody (diluted 1:500) as described (Esaki et al., 2009).

### Semi-quantification of gene expression levels by RT–PCR

Approximately 20 fertilized embryos reared in 0.006% (0.7 mM Na^+^) salt water were dechorionated at 24 hpf, rinsed with 0.0003% (0.03 mM Na^+^) or 0.6% (70 mM Na^+^) salt water, and cultured in 50 ml of 0.0003 or 0.6% salt water in a 100-mm Petri dish for 6 days (=7-dpf), respectively. As a control, dechorionated embryos were continuously cultured in 0.006% salt water until 7-dpf. Living larvae in a dish were collected and total RNA was extracted, and then single-stranded cDNA was prepared as previously described (Hoshijima and Hirose, 2007). The cDNA fragments of *ca2a*, *ca15a*, *ribosomal protein L9* (*rpl9*) were amplified by PCR in 10-μl reaction volumes containing 1 μl cDNA, 5 μl 2 × GoTaq Green Master Mix (Promega), and 0.3 μM each of forward and reverse primers. Following denaturation for 3 min at 94°C, amplification was performed by 25 and 30 cycles of 15 s at 94°C, 20 s at 60°C, and 2 min at 72°C.

### Real-time PCR (qPCR)

Total RNA used for qPCR was extracted independently of that used for reverse transcription-polymerase chain reaction (RT-PCR) from 20 6-dpf larvae adapted for 4 days to 0.0003, 0.006, and 0.6% salt water by the acid guanidine isothiocyanate-phenol-chloroform method with Isogen (Nippon Gene Co., Toyama, Japan). Synthesis of first strand cDNA and qPCR were performed as described previously (Hoshijima and Hirose, 2007). The primer sets used for qPCR were designed according to Lin et al. (2008) and have the following sequences (in the order of sense and antisense): *ca2a*, 5′-tcgtctgcaaacagtccatca-3′ and 5′-tccgagaggcccttcattt-3′; *ca15a*, 5′-tcagaacacactgtggatggc-3′ and 5′-tgcttgcttcattcgttccc-3′; and β*-actin*, 5′-attgctgacaggatgcagaag-3′ and 5′-gatggtccagactcatcgtactc-3′, which have Tm of 56/55, 58/55, and 56/60°C, respectively.

### 5′-Rapid amplification of cDNA end (RACE) and morpholino injection

To obtain the accurate sequence around the translation start site on the *ca2a* and *ca15a* genes, 5′-RACE was performed with First Choice RLM-RACE Kit (Ambion) according to the manufacturer’s instruction. The following gene-specific reverse primers were used: for *ca2a*, 5′-atttgccattgaccgtgtgt-3′ (nt 350–369), 5′-agtctgaatgtgcctgtgacc-3′ (nt 281–301), and 5′-gagtgtccattgttctggatgt-3′ (nt 205–226) and for *ca15a*, 5′-ccccagtgaagatgaaagtgtt-3′ (nt 418–439), 5′-atatgaaggtggtgttggcatc-3′ (nt 303–324), and 5′-cagtaatgtggagcaagttcagg-3′ (nt 192–214). Antisense Morpholino oligonucleotides specific for *ca2a* and *ca15a* genes (*ca2a*-MO and *ca15a*-MO) were synthesized by Gene Tools. The Morpholino oligonucleotides were designed to complementarily anneal to mRNA around the translation start site: *ca2a*-MO, 5′-ccattccgccagctgtgctgcagtc-3′ and *ca15a*-MO, 5′-tcatctcttcgacttgtttctccag-3′. Approximately 1 nl of 1 mg/ml *ca2a*-MO or *ca15a*-MO in 1× Danieau buffer [58 mM NaCl, 0.7 mM KCl, 0.4 mM MgSO_4_, 0.6 mM Ca(NO_3_)_2_, 5 mM HEPES, pH 7.6] was injected into the yolk of one-cell embryos as described (Hoshijima et al., 2002) and they were reared in 0.006% (0.7 mM Na^+^) salt water.

### Na^+^ accumulation analysis

Na^+^ accumulation analyses were performed with Sodium Green tetra-acetate cell permeant (Invitrogen) as described (Esaki et al., 2007) using 55-hpf embryos and FITC optics of a Zeiss Axiovert 200 inverted fluorescence microscope. It is essential for obtaining clear images to raise, maintain, and anesthetize embryos in 0.006% salt water (fish water described in The Zebrafish Book) and to observe the fluorescence with a high-magnification objective lens in order to avoid interference by background fluorescence from the yolk; after 3 dpf, the swimbladder begins to develop and emits strong interfaring fluorescence. Single focused images were created from a series of partially focused images with HeliconFocus software (Helicon, Ukraine). The pH of the incubation media (0.0003 and 0.6% salt water) was adjusted to 6.8–7.2. For measuring the effect of gramicidin, larvae were treated with 10 μM gramicidin (stock solution: 2 mM in dimethyl sulfoxide) for 30 min at 25°C before the addition of Sodium Green.

### Immunohistochemistry

Immunohistochemistry with zebrafish larvae at 2 dpf and 7 dpf was performed as described previously (Esaki et al., 2007), with exceptions that larvae were fixed in 4% paraformaldehyde in PBS for 1 h and dehydrated in 100% methanol for 10 min at −20°C. For double staining with concanavalin A (ConA), the larvae were incubated with 50 μg/ml of Alexa Fluor 594-conjugated ConA for 30 min prior to fixation. Incubation time with antibodies was also modified to 40 h at 4°C with rabbit anti-zCA2a antiserum (diluted 1:500 with PBS containing 0.1% Tween 20 and 10% sheep serum), rabbit anti-zCA15a antiserum (diluted 1:500), and rat anti-dace H^+^-ATPase antiserum (diluted 1:500).

Immunohistochemistry of gill sections were performed as described previously (Nakada et al., 2007) with the exception that gills from adult zebrafish were fixed with 2% paraformaldehyde in PBS at 4°C for 1 h. After blocking with FBS, the sections were incubated with rabbit anti-zCA2a antiserum (diluted 1:1,000 with PBS containing 5% FBS), rabbit anti-zCA15a antiserum (diluted 1:1,000), rat anti-eel Na^+^/K^+^-ATPase antiserum (diluted 1:1,000) for two overnights at 4°C. In the case of immunostaining with transgenic zebrafish HG9B gills, anti-GFP monoclonal antibody (diluted 1:100, Clontech, Palo Alto, CA, USA) was used. Following a wash with PBT, the larvae were further incubated for 2 h at room temperature with Alexa Fluor 488-conjugated anti-rabbit IgG (diluted 1:1,000, Invitrogen), Alexa Fluor 594-conjugated anti-rabbit IgG (diluted 1:1,000, Invitrogen), Alexa Fluor 594-conjugated anti-rat IgG (diluted 1:1,000, Invitrogen), or Alexa Fluor 488-conjugated anti-mouse IgG (diluted 1:1,000, Invitrogen). Additionally, Hoechst 33342 (100 ng/ml; Invitrogen) was added in the secondary antibody solutions for nuclei staining. Fluorescence images were acquired by using Axiovert 200 M epifluorescence microscope (Carl Zeiss, Thornwood, NY, USA) equipped with an ApoTome optical sectioning device (Carl Zeiss).

### *In situ* proximity ligation (PLA) assay

Adult zebrafish gill sections were prepared as described above, and were blocked with 5% FBS in PBS for 1 h at room temperature. The sections were incubated with rat anti-zNhe3b (diluted 1:1,000) and rabbit anti-zCA2a (diluted 1:1,000) or rabbit anti-zCA15a (diluted 1:1,000) or rabbit anti-zRhcg1a (diluted 1:1,000) for one overnight at room temperature, followed by corresponding secondary antibodies conjugated with the PLA oligonucleotide probes (PLA probe rabbit PLUS and PLA probe rat MINUS; Olink Bioscience, Uppsala, Sweden; diluted 1:5 with 5% FBS in PBS) for 2 h at 37°C. Hybridization and ligation of the connector oligonucleotides, a rolling-circle amplification, and detection of the amplified DNA products were performed with *in situ* PLA detection kit 613 (Olink Bioscience) according to the manufacturer’s instruction. Alexa Fluor 488-conjugated anti-rat IgG (diluted 1:1,000, Invitrogen) was added in order to visualize zNhe3b positive cells. Following addition of 0.1% *p*-prenylenediamine and 90% glycerol in PBS, the slides were covered with cover slips and photographed in the same manner described above.

## Results

### Expression of zebrafish CA2a and CA15a proteins IN H-MRC

**Figure A1:**
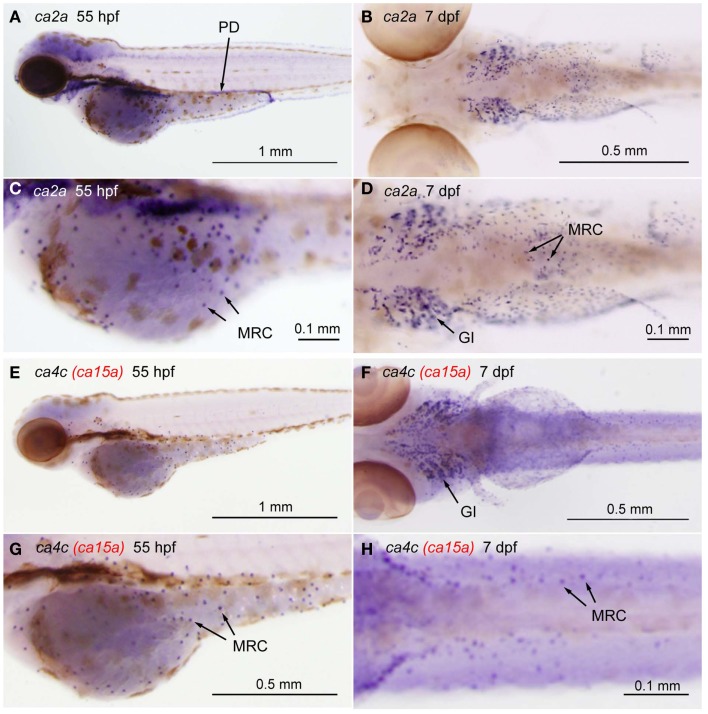

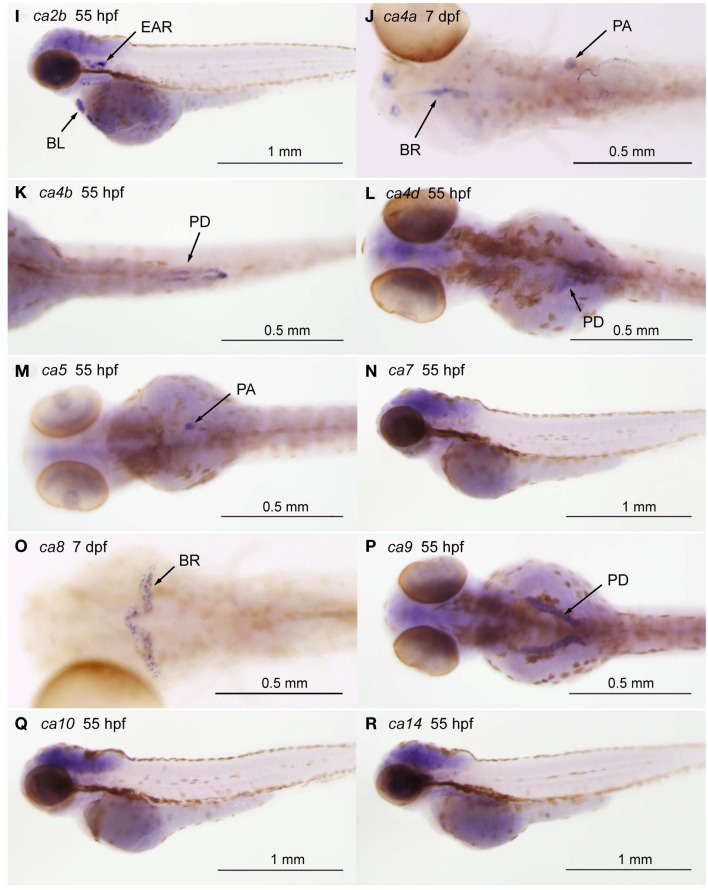
**Tissue-specific expression of CA isoforms in zebrafish larvae**. Expression of *ca* genes was analyzed in 55-hpf and 7-dpf larvae by whole mount *in situ* hybridization with cRNA probes against *ca2a* [**(A,C)**, lateral views and **(B,D)**, ventral views], *ca2b* [**(I)**, lateral view], *ca4a* [**(J)**, dorsal view], *ca4b* [**(K)**, ventral view], *ca4c* or *ca15a* [**(C,E)**, lateral views and **(D,F)**, ventral views], and *ca4d* [**(L)**, dorsal view], *ca5* [**(M)**, dorsal view], *ca7* [**(N)**, lateral view], *ca8* [**(O)**, dorsal view], *ca9* [**(P)**, dorsal view], *ca10* [**(Q)**, lateral view], and *ca14* [**(R)**, lateral view]. **(C,D,G,H)** Magnified views of **(A,B,E,F)**, respectively. Tissue- or cell-specific expression of individual *ca* genes was indicated with arrows in each panel. Abbreviations are gill (GI), mitochondria-rich cell (MRC), blood cell (BL), brain (BR), pronephric ducts (PD), pancreas (PA). We note that broad staining in the brain was non-specific background as it was observed with sense cRNA probes. Four CA4 isoforms predicted from zebrafish database showed different tissue-specific distribution. *ca4a*, most homologus to mammalian *CA IV*, is expressed in the pancreas and expected to be involved in the regulation of the luminal pH of the pancreatic duct system (Fujikawa-Adachi et al., 1999). The other two isoforms *ca4b* and *ca4d* are expressed respectively in the posterior part and anterior part of the renal pronephric ducts. These expressions reminds that localization of mammalian membrane-bound CA in the proximal tubule cells and the intercalated cells of the collecting ducts, which is suggested to contribute to reabsorption of ${\text{HCO}}_{3}^{-}$ and regulation of acid-base balance of urine (Dobyan and Bulger, 1982). Its location suggests a role in regenerating CO_2_, a substrate for CA II. For *ca4c* (i.e., *ca15a*), see the text.

The previous study by Lin et al. (2008) has analyzed the expression of 10 zebrafish *ca* genes, i.e., *ca2* (*zca2-like a*), *cah-z* (*zca2-like b*), *ca4a*, *ca5*, *ca6*, *ca7*, *ca9*, *ca10a*, *ca14*, and *ca15a*, in zebrafish embryo, and has demonstrated that only two isoforms, *ca2a* and *ca15a*, are specifically expressed in the skin and gill H-MRCs, ionocytes rich in H^+^-ATPase. We also independently analyzed the expression of 12 zebrafish *ca* isoforms [*ca2* (*ca2a*), *cah-z* (*ca2b*), *ca4a*, *ca4b*, *ca4d*, *ca5*, *ca7*, *ca8*, *ca9*, *ca10*, *ca14*, and *ca15a*] in 55-hpf and 7-dpf larvae by whole mount *in situ* hybridization. Consistent with the findings by Lin et al. (2008), only *ca2a* and *ca15a* expression was found to occur sporadically on the yolk sac surface and the gills (Figure A1a in Appendix). In addition, *ca2a* expression was also detected in the pronephric ducts, gut, and muscle (Figure A1a in Appendix). As summarized in Figure A1b in Appendix, other 7 *ca* isoforms (*ca2b*, *ca4a*, *ca4b*, *ca4d*, *ca5*, *ca8*, and *ca9*) showed tissue-specific expression patterns; we were not able to observe specific expression of *ca7*, *ca10*, or *ca14* by whole mount *in situ* hybridization.

**Figure 1:**
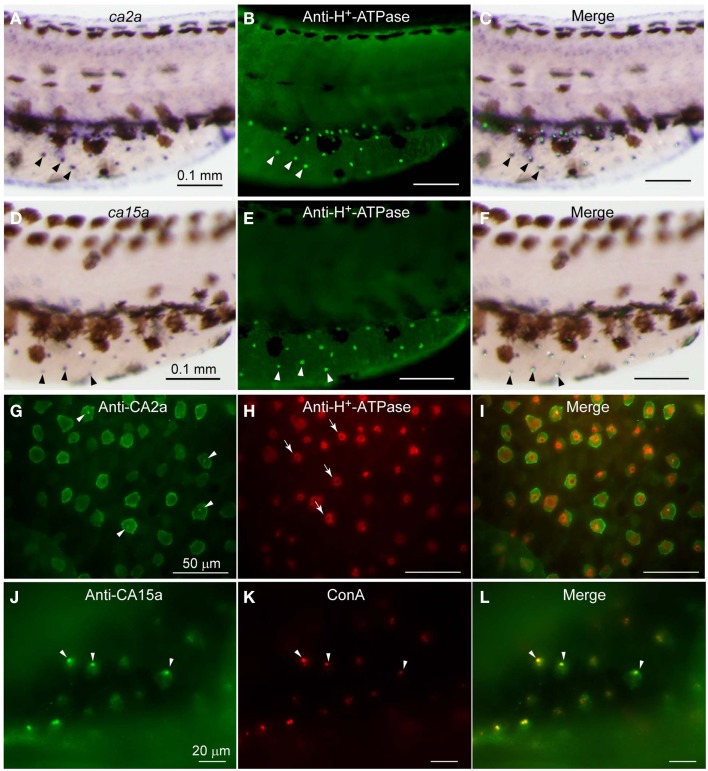
**Expression of *ca2a* and *ca15a* mRNA and their protein products in H-MRC**. Zebrafish larvae of 55-hpf were probed for expression of the indicated genes **(A,D)** and their products **(G,J)**, and the types of positive cells were identified by costaining with anti-H^+^-ATPase **(B,E,H)** and concanavalin A [ConA, **(K)**], which are markers for H-MRC. Right panels **(C,F,I,L)** are merged images. All panels show lateral views of dorsal trunk and yolk sac extension of specimens oriented dorsal side up, anterior to the left. Large (30–50 μm) dendritic cells of dark brown in **(A,C,D,F)** are melanocytes. Arrowheads in **(A–F)** point to typical H-MRCs coexpressing *ca2a*, *ca15a*, and H^+^-ATPase. Arrowheads in **(G,J–L)** indicate the apical pits of H-MRC. Arrows in **(H)** show donut-like staining surrounding the pits.

As expected from the similar distribution patterns of *ca2a* and *ca15a* mRNA revealed by the above *in situ* hybridization analysis, their possible colocalization in H-MRC was examined by double staining for CA’s (*in situ* hybridization) and markers specific for subpopulations of MRCs (immunohistochemistry). When zebrafish larvae that had been stained with anti-H^+^-ATPase (Figures 1B,E) were further incubated with *ca2a* and *ca15a*cRNA probes (Figures 1A,D), clear colocalization of *ca2a* and H^+^-ATPase (Figure 1C) and *ca15a* and H^+^-ATPase (Figure 1F) was observed.

**Figure 2:**
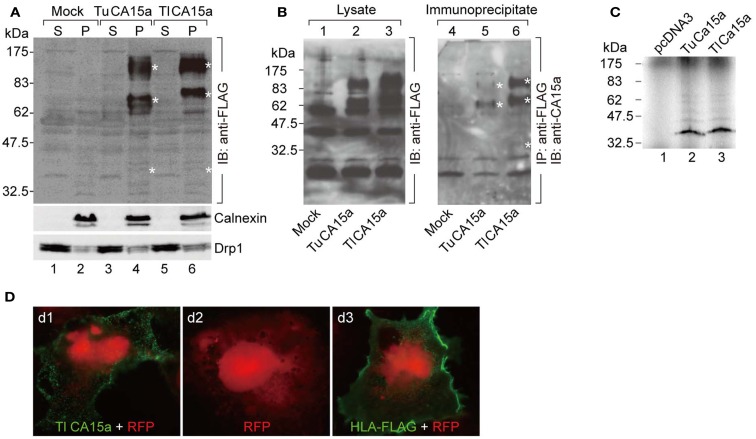
**Cell surface expression of CA15a and specificity of anti-CA15a**. **(A)** Membrane localization and oligomeric nature of zebrafish CA15a synthesized in cultured cells. 293T cells were transfected with mock, TuCA15-FLAG, or TlCA15-FLAG (Tu for Tuebingen wildtype line and Tl for Tupfel long fin wildtype line, which exhibited a slight variation in their amino acid sequences as mentioned in Section “Materials and Methods”. Two days after transfection, the cells were fractionated into the cytosol (S) and membrane pellets (P). The fractions (25 μg of proteins) were analyzed by Western blotting with antibodies against FLAG (top panel), calnexin (an ER membrane marker protein; middle panel), and Drp1 (a member of the dynamin family of large GTPases; bottom panel). **(B)** Antibody specificity. 293T cells were transiently transfected with mock (lanes 1 and 4), TuCA15-FLAG (lanes 2 and 5), or TlCA15-FLAG (lanes 3 and 6). Cells were homogenized in lysis buffer containing 1 M urea and 1 mM dithiothreitol (see Materials and Methods). The lysates were subjected to immunoprecipitation with anti-FLAG antibody beads followed by Western blotting with anti-CA15a antibody (lanes 4–6). The lysates (40 μg of proteins) were also analyzed by Western blotting with anti-FLAG antibody (lanes 1–3). Asterisks indicate the bands corresponding to monomer, dimer, and tetramer of CA15-FLAG. **(C)** TuCA15-FLAG (lane 2) and TlCA15-FLAG (lane 3) were translated *in vitro* in the presence of [^35^S]methionine and [^35^S]cysteine, and were then analyzed by SDS–PAGE followed by autoradiography. **(D)** Cell surface expression of CA15a demonstrated by immunofluorescence. COS7 cells were transfected with red fluorescent protein (RFP, red) alone (d2) or along with either TlCA15-FLAG (d1) or HLA-FLAG (d3). Non-permeabilized cells were analyzed by immunofluorescence microscopy with anti-FLAG antibody (green). HLA (human leukocyte antigen) or MHC (major histocompatibility complex) is a cell surface molecule.

**Figure A2:**
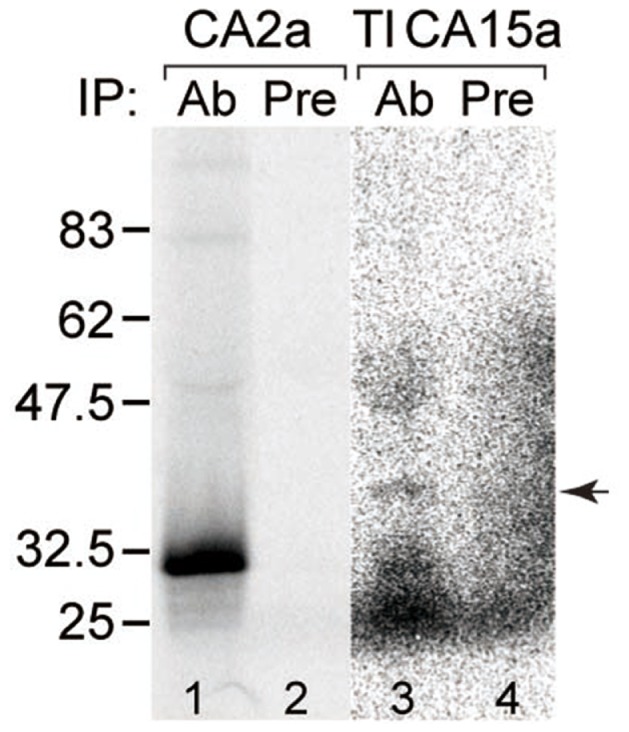
**Specificity of antisera**. Recognition of the antigens by the corresponding antisera demonstrated by immunoprecipitation. CA2a (lanes 1 and 2) and TlCA15-FLAG (lanes 3 and 4) were translated *in vitro* in the presence of [^35^S]methionine and [^35^S]cysteine. The labeled proteins were subjected to immunoprecipitation with anti-CA2a antiserum (lane 1, Ab), anti-TuCA15a antiserum (lane 3, Ab), or corresponding preimmune serum (lanes 2 and 4, Pre). The immunoprecitates were analyzed by SDS–PAGE followed by autoradiography. Arrow indicates the position of the band corresponding to TlCA15-FLAG, indicating crossreactivity of anti-TuCA15a with TlCA15a.

To further confirm the colocalization of CA2a and CA15a in H-MRCs at the protein level, we generated rabbit polyclonal antiserum against CA2a and rabbit peptide antiserum against CA15a. These antisera specifically recognized either CA2a or CA15a proteins by immunoprecipitation (Figure A2 in Appendix) and immunostaining of COS7 cells exogenously expressing zebrafish CA15a (Figure 2D). Next, we performed immunohistochemistry of 7-dpf zebrafish larvae with anti-CA2a or anti-CA15a antiserum in combination with H-MRC markers: anti-H^+^-ATPase, and ConA (van der Heijden et al., 1997; Lin et al., 2006; Esaki et al., 2007). As shown in Figures 1G–L, signals for CA2a and CA15a were detected in H-MRCs on the yolk sac.

### Extracellular surface localization of CA15a in H-MRC

**Figure A3:**
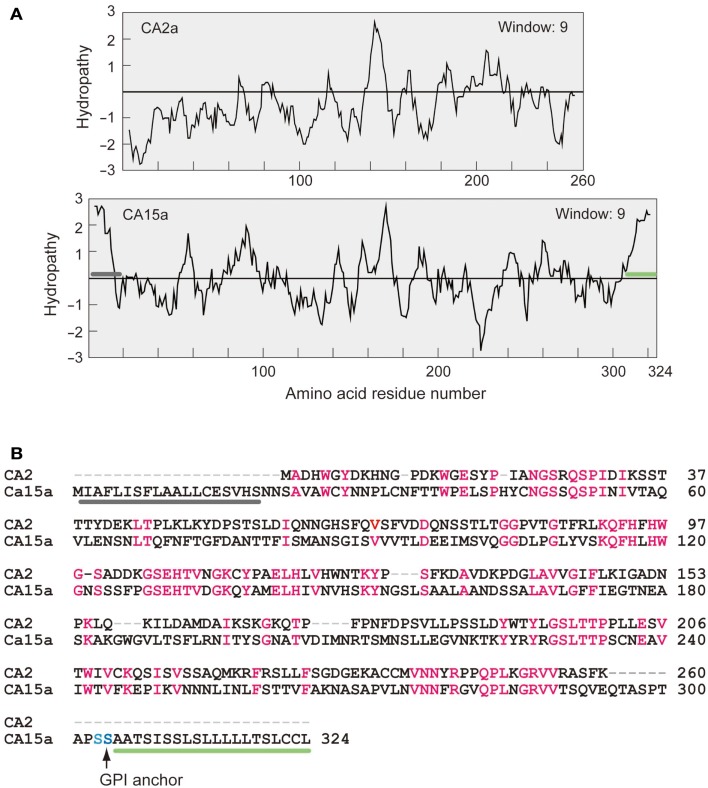
**Hydropathy profiles (A) and amino acid alignment (B) of zebrafish CA2a and CA15a**. **(A)** Kyte–Doolittle hydropathy plots generated by using a window size of 9 (Kyte and Doolittle, 1982). Gray bar represents a putative signal peptide and green bar, a hydrophobic glycosylphosphatidylinositol (GPI)-anchor signal. **(B)** Comparison of amino acid sequences of zebrafish CA2a and CA15a. Identical residues are highlighted in red. GPI anchor site is indicated by a vertical arrow. Gray and green bars correspond to those in **(A)**.

It is known that mammalian CA II is expressed as a soluble cytosolic protein, and mammalian CA IV is secreted and retained on the plasma membrane *via* the C-terminal glycosylphosphatidylinositol (GPI) anchor (Breton, 2001; Schwartz, 2002; Purkerson and Schwartz, 2007). Hydropathy plot analysis predicted that zebrafish CA2a is a soluble protein with no apparent hydrophobic stretch (Figure A3 in Appendix) and that CA15a possesses an N-terminal hydrophobic region which would function as a signal sequence (Figure A3 in Appendix). In addition, zebrafish CA15a is closely related to mammalian CA IV and contains a potential GPI anchor site at the C terminus (Lin et al., 2008) (Figure A3 in Appendix and also see Discussion). To determine the subcellular distribution of zebrafish CA15a, we performed subcellular fractionation of 293T cells transiently transfected with FLAG-tagged CA15a. Western blotting with anti-FLAG antibody revealed that CA15a-FLAG was detected in the membrane fraction (Figure 2A). The Western blotting detected a weak band of ∼38 kDa corresponding to the monomeric form and two very strong bands with molecular masses of approximately 70 and 140 kDa, which were much higher than the calculated one (∼35 kDa). A similar pattern of Western blotting was also observed by immunoprecipitation with anti-FLAG followed by immunostaining with anti-CA15a (Figure 2B). The higher molecular weight species were present as major forms even in the presence of 6 M urea and 10 mM dithiothreitol (data not shown). When CA15a-FLAG constructs were *in vitro* translated in the presence of [^35^S]methionine and [^35^S]cysteine, ^35^S-labeled proteins were detected as single bands at ∼35 kDa (Figure 2C). These results suggest that CA15a-FLAG forms very stable oligomeric structures.

Next, to determine whether CA15a is expressed extracellularly, we performed immunofluorescence staining of COS7 cells, exogenously expressing FLAG-tagged zebrafish CA15a, without membrane permeabilization. The anti-FLAG antibody showed a staining pattern of membrane proteins (Figure 2D), a pattern very similar to that of HLA, indicating that CA15a is present on the extracellular side of the plasma membrane.

### Salinity-dependent expression of CA2a and CA15a in zebrafish larvae

**Figure 3:**
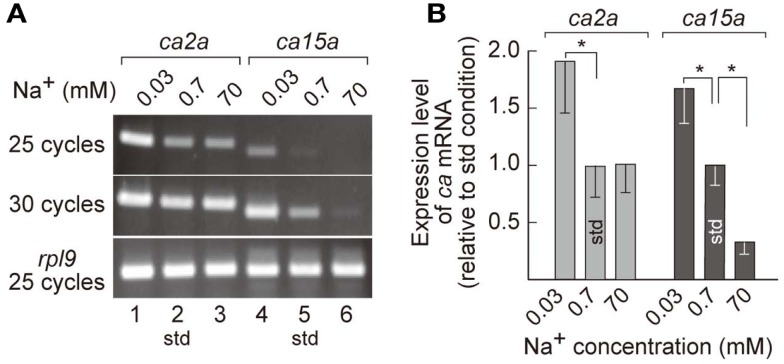
**Salinity-dependent expression of *ca2a* and *ca15a* in zebrafish larvae**. **(A)** Expression level of *ca2a* and *ca15a* in different salinity medium was analyzed by RT-PCR with RNA extracted from 7-dpf larvae reared in 0.0003% (0.03 mM Na^+^), 0.006% (0.7 mM Na^+^), or 0.6% (70 mM Na^+^) salt water. *rpl9* (ribosomal protein L9 mRNA) was used as expression control. **(B)** Levels of *ca2a* and *ca15a* mRNA expression estimated by quantitative RT-PCR (qPCR) (*n *= 4). Data are expressed relative to the value obtained under the standard culture conditions (std; 0.7 mM Na^+^). *p < 0.05 in the comparison with the standard culture condition (Student’s *t*-test).

MRCs of FW fishes have been presumed as primary sites for ion uptake from FW. Indeed, we have demonstrated that ambient Na^+^ was absorbed in H-MRCs (Esaki et al., 2007). Furthermore, efficiency of ion uptake was dependent on ambient salinity (Boisen et al., 2003), suggesting that gene regulation in MRCs is required for appropriate ion uptake in different salinity environment. To identify the genes whose expression is correlated with ambient salt concentrations, we prepared mRNA preparations from 7-dpf zebrafish larvae acclimated to 0.0003% (0.03 mM Na^+^), 0.006% (0.7 mM Na^+^; the standard culture condition), and 0.6% (70 mM Na^+^) salt water, performed RT-PCR, and found that expression levels of *ca2a* in 0.03 mM Na^+^ water substantially increased compared to those in 0.7 mM Na^+^ and 70 mM Na^+^ water (Figure 3A). On the other hand, expression levels of *ca15a* were more strictly dependent on ambient salinity; those were elevated in 0.03 mM Na^+^ but reduced in 70 mM Na^+^ compared to those in 0.7 mM Na^+^ water. The salinity-dependent expression of the *ca* genes in larvae was confirmed by real-time PCR (Figure 3B). Thus, expression of *ca2a* and *ca15a* was regulated by ambient salinity concentration.

### Inhibition of Na^+^ uptake by CA2a and CA15a depletion

**Figure 4:**
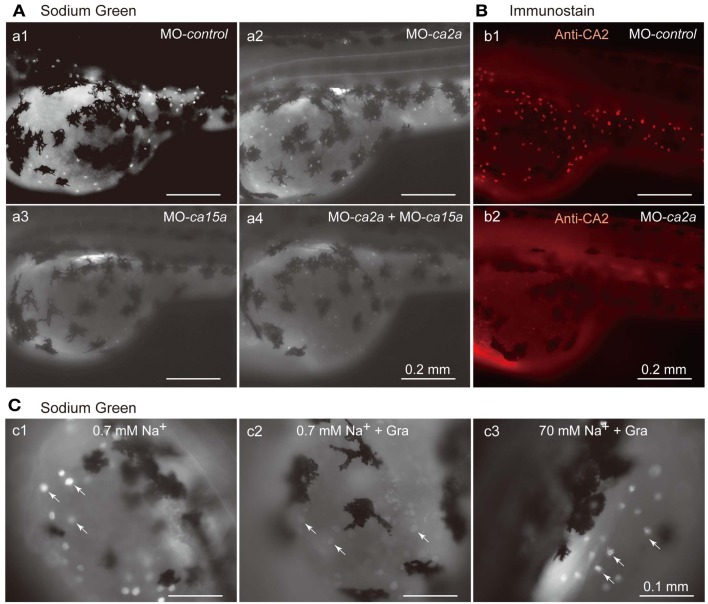
**Inhibitory effects of antisense *ca2a* and *ca15a* MOs on Na^+^ accumulation in MRC**. **(A)** Na^+^ accumulation was analyzed at 55-hpf by monitoring Sodium Green fluorescence (white spots) in a larva injected with 1 ng each of *control*-MO (a1), *ca2a*-MO (a2), *ca15a*-MO (a3), or *ca2a*-MO plus *ca15a*-MO (a4). Endogenous fluorescence from the yolk is seen as a weak background. Relatively strong background on the dorsal area of the yolk is due to the formation of swimbladder, which becomes evident at 55–60 hpf. Large and dendritic dark cells are melanocytes. **(B)** Confirmation of depletion of the CA2a protein in morphants treated with *ca2a*-MO. Larvae were treated with *control*-MO (b1) and *ca2a*-MO (b2) and stained with anti-CA2a antiserum. **(C)** Sodium Green fluorescence reflecting intracellular concentrations of Na^+^. Zebrafish larvae were stained with Sodium Green with or without pretreatment with gramicidin (Gra) in the standard culture medium (c1 and c2) and in a high salt medium (c3). Gramicidin was added to equilibrate the intracellular and extracellular Na^+^ concentrations.

Restricted expression of CA2a and CA15a in H-MRCs, where Na^+^ uptake occurs (Esaki et al., 2007), leads to a possibility that these CA isoforms are involved in Na^+^ uptake in H-MRCs. To elucidate the possibility, we depleted CA2a and/or CA15a from zebrafish larvae by Morpholino antisense oligonucleotides (MO) injection and examined Na^+^ uptake ability in the larvae with Sodium Green, a Na^+^-dependent fluorescent reagent, which has been demonstrated to be useful for detecting Na^+^ absorbed in MRCs (Esaki et al., 2007). Injection of control-MO exerted no significant effects on the Sodium Green signal (Figure 4Aa1). MO-induced depletion of CA2a and CA15a was confirmed by immunostaining. A typical result of MO-*ca2* is shown in Figure 4B; a similar result was also obtained for MO-*ca15a* (data not shown). When CA2a was depleted, fluorescence intensity of Sodium Green in MRC was significantly reduced, indicating that CA2a significantly contributes to Na^+^ uptake in MRC (Figure 4Aa2). Also in CA15a-depleted larvae, Na^+^ uptake activity was markedly reduced (Figure 4Aa3). Simultaneous depletion of CA2a and CA15a did not show any additive effect as the Na^+^ uptake activity was similar to that in CA15a-depleted larvae (Figure 4Aa4). These results indicate that CA15a is also required for efficient Na^+^ uptake in MRCs although contribution of CA2a and CA15a to Na^+^ uptake is not additive.

In an attempt to quantify the Na^+^ concentration in H-MRCs, we treated 55-hpf larvae with gramicidin, an Na^+^-permeable pore-forming antibiotic, to equilibrate the intracellular Na^+^ with extracellular Na^+^. As expected, in a low salt medium (0.7 mM Na^+^), intracellular Na^+^ was depleted in the presence of gramicidin and Sodium Green signals became weak (Figure 4Cc2). In contrast, in a high Na^+^ medium (70 mM), Sodium Green fluorescence remained relatively strong (Figure 4Cc3). We next tried to draw a calibration curve by changing the Na^+^ concentration of the culture medium, but obtaining an accurate curve was somehow difficult because of variabilities in background fluorescence from the yolk and in viability of larvae in the presence of gramicidin, which requires a relatively long incubation for pore formation.

### Apical membrane localization of Nhe3b in H-MRC

**Figure 5:**
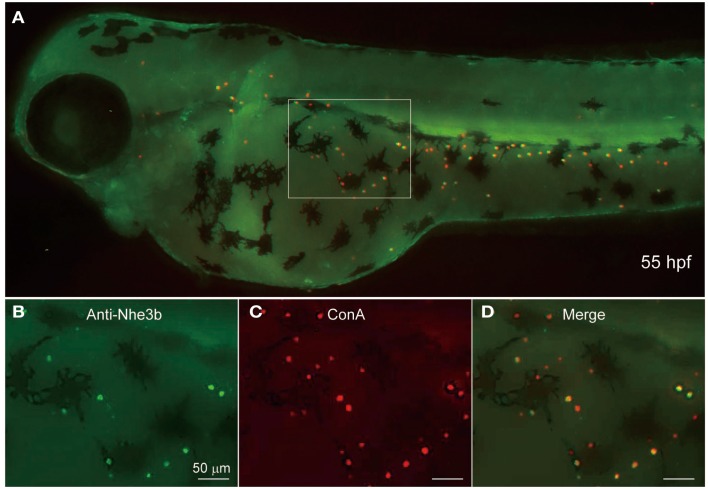
**Localization of Nhe3b in H-MRCs of larval skin of zebrafish**. **(A)** A zebrafish larva stained with anti-Nhe3b (green) and ConA (red), a marker for apical membrane of H-MRC. **(B–D)** Enlarged views of boxed area in **(A)**. Green signals for Nhe3b were relatively weak and therefore enhanced before merging with red signals for ConA. hpf, hours postfertilization.

Yan et al. (2007) showed by using non-homologous antiserum (anti-dace Nhe3) that Nhe3b is expressed in H-MRC of the zebrafish gill. To extend this observation to the skin MRCs, we stained 55-hpf larval skin of zebrafish by using a homologous antiserum (anti-zebrafish Nhe3b). A clear staining of the apical region of H-MRCs was observed (Figure 5); apical membrane staining was confirmed by costaining with ConA, which is known to label the apical region of MRCs of fishes (van der Heijden et al., 1997; Sakamoto et al., 2000; Lin and Hwang, 2004; Evans et al., 2005; Lin et al., 2006). Among ConA-positive H-MRCs, only certain populations were positive for Nhe3b (Figures 5B,C). Whether this represents a subpopulation of H-MRCs or just a difference in the Nhe3b levels remains to be determined.

### Close association of Nhe3b with CA2a, CA15a, and Rhcg1 in H-MRC revealed by *in situ* PLA

In addition to the above-mentioned colocalization of Nhe3b, CA2a, and CA15a whose mRNA levels have been shown to be upregulated on acclimation to low Na^+^ FW (this study) (Yan et al., 2007; Lin et al., 2008), we previously reported that the ammonia transporter Rhcg1 is also expressed in apical membrane of zebrafish H-MRC in a salinity-dependent manner (Nakada et al., 2007).

**Figure 6:**
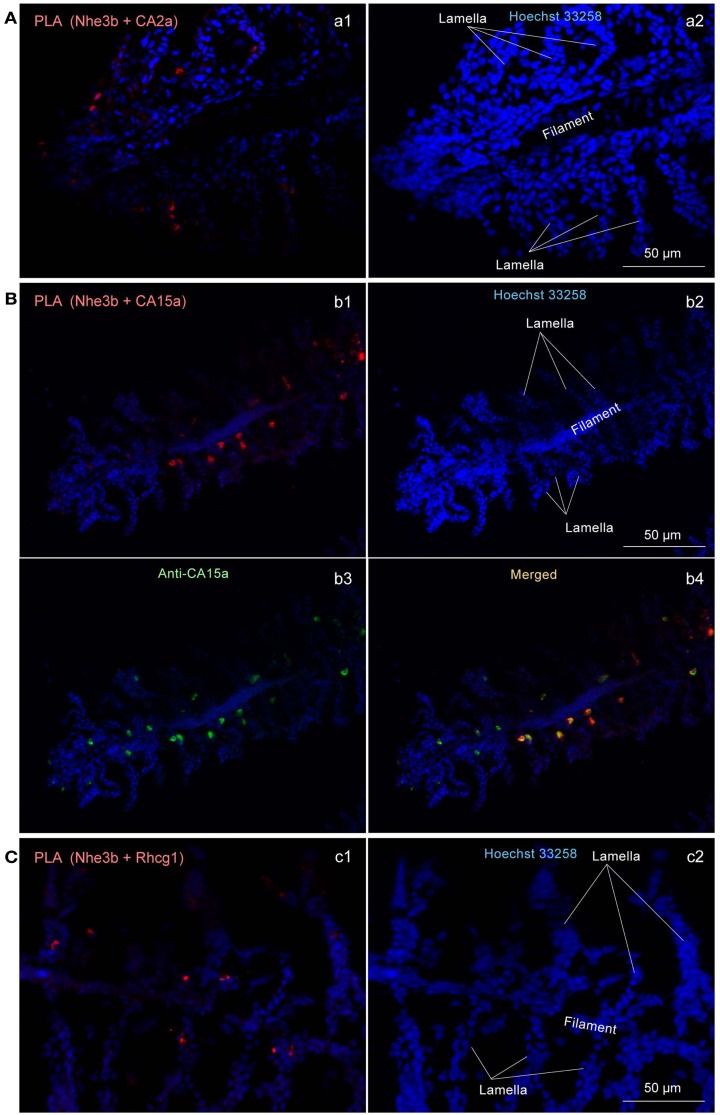
**Close association of CA2a, CA15a, Nhe3b, and Rhcg1 visualized by *in situ* proximity ligation immunoassay (PLA)**. **(A–C)** Zebrafish gills were fixed with 4% PFA, incubated with two primary antibodies (a1: rabbit anti-CA2a and rat anti-Nhe3b; b1: rabbit anti-CA15a and rat anti-Nhe3b; and c1: rabbit anti-Rhcg1 and rat anti-Nhe3b), and processed for detecting protein-protein interaction *in situ* with a Duolink assay kit. Each red signal represents an interaction detected by the kit based on proximity-dependent DNA ligation. (b3) Simultaneous visualization of CA15a by using Alexa Fluor 488-conjugated anti-rat IgG as secondary antibody (green). Blue signals representing nuclei were enhanced in a2, b2, and c2 to show the gill structure. Red PLA signals were enhanced in a1, and b1 but not in c1.

**Figure A4:**
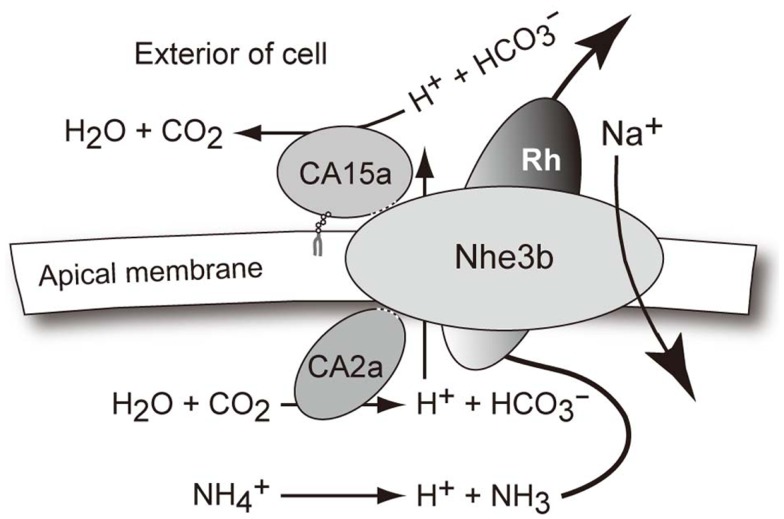
**A hypothetical model for the interaction of CA2a, CA15a, Nhe3b, and Rhcg1 acting together as a transport metabolon or transportsome to take up Na^+^ in freshwater**. Concerning the Na^+^ uptake by zebrafish in FW conditions, it seems plausible that at the apical side of H-MRC, Na^+^ is taken up by sodium/proton exchanger Nhe3b in exchange for H^+^, which is generated by carbonic anhydrase CA2a. The excreted H^+^ is then titrated by CA15a. Although not shown in the transport metabolon model, H^+^ may also be secreted *via* H^+^-ATPase. The net reaction results in accumulation of Na^+^ and ${\text{HCO}}_{3}^{-}$, as typically seen in the mammalian renal proximal tubule cells for reabsorption of bicarbonate and sodium ions (Shirley and Unwin, 2005; Purkerson and Schwartz 2007; Bobulescu and Moe 2009). Concerning the major driving force, however, the two systems must be quite different since the local ionic gradients are much less favorable for Na^+^/H^+^ exchange in the case of FW fish H-MRCs whose apical membrane is energized by H^+^-ATPase (Harvey and Wieczorek, 1997; Nakhoul and Hamm 2002). Extra H^+^ generated from ${\text{NH}}_{4}^{+}$ may act as a part of driving force for Nhe3b.

To determine whether Nhe3b, Rhcg1, and the CA isoforms identified above form a transport metabolon, a closely associated complex of proteins that facilitates transport of ions, we performed a recently developed immunoassay, termed the PLA (Fredriksson et al., 2002; Esaki et al., 2009), which makes it possible to visualize protein interactions using standard fluorescence microscopy. *In situ* PLA signals were detected on gill sections at inter-lamellar regions among the combinations of CA2a and Nhe3b (Figure 6Aa1), CA15a and Nhe3b (Figure 6Bb1), and Rhcg1 and Nhe3b (Figure 6Cc1), suggesting a very close association of Nhe3b with CA2a, CA15a, and Rhcg1 in zebrafish H-MRCs. We used zebrafish gill sections instead of larval yolk sac membranes because the former gave much stronger signals predominantly at the bases between the lamellae (Figure 6). The PLA signal of Nhe3b/Rhcg1 was relatively strong and could be seen without signal enhancement (Figure 6Cc1), but those of Nhe3b/CA2a (Figure 6Aa1) and Nhe3b/CA15a (Figure 6Bb1) were weak, suggesting a tight association of Nhe3b and Rhcg1, and weak but substantial interactions among Nhe3b, CA2a, and CA15a (Figure A4 in Appendix).

## Discussion

In the present study, we demonstrated the presence of a CA2a/CA15a/Nhe3b/Rhcg1 transport metabolon that is expected to play a central role in absorbing Na^+^ against the uphill gradient by FW fishes. The concept of metabolon was first proposed for a complex of enzymes that catalyze a series of reactions in a metabolic pathway and later extended to complexes of transporters and signaling cascades. Because of the limitation of the method we used (PLA), it is not clear if the components are directly associated or they are just clustered at the plasma membrane of H-MRCs of zebrafish. In any case, the term “transport metabolon” can be used since metabolon is a functional unit not necessary implying direct physical association. Concerning the transport metabolons involving CA, Vince and Reithmeier (1998) first demonstrated a CAII/AE1 transport metabolon in the erythrocyte membrane. It is, however, still debated about their physical association (Boron, 2010).

In fish, Nhe3 has attracted a great deal of attention for its possible involvement in osmoregulation (NaCl absorption) and acid-base regulation (H^+^ extrusion) in FW since its first cloning of a fish counterpart in Osorezan dace (Hirata et al., 2003). In our working model (Figure A4 in Appendix), the major player is Nhe3b, which is supposed to be an electroneutral exchanger like Nhe1. This electroneutral nature, although not confirmed by electrophysiological measurements, poses a difficult question regarding the driving force for the uphill Na^+^ absorption. One possibility is an alteration of coupling ratio of Na^+^ to H^+^ from 1:1 to 2:1 or more when Nhe3b is incorporated into the transport metabolon. This could be an interesting issue to be addressed in future research. A similar but more complex transport metabolon has been proposed, in a review article by Wright and Wood (2009), as “Na^+^/NH_4_^+^ exchange complex.” Both models are consistent with the previous studies by (i) Lin et al. (2008) who identified a H-MRC-specific CA isoform, CA15a, and demonstrated its functional link to the Na^+^ uptake in the skin of zebrafish larvae, (ii) Yan et al. (2007) who demonstrated the presence of Nhe3b in H-MRC of the zebrafish gill, (iii) Hirata et al. (2003) who proposed a Na^+^ uptake model based on a functional coupling of CA2 and Nhe3 through the analysis of Osorezan dace living in an acidic lake, (iv) Nakada et al. (2007) on the ammonia transporter Rhcg1 in zebrafish H-MRCs, which suggested a link between ammonia excretion and Na^+^ uptake, (v) Kumai and Perry (2011) who demonstrated that ammonia excretion *via* Rhcg1 facilitates Na^+^ uptake in larval zebrafish in acidic water, and (vi) Shih et al. (2012) on zebrafish Rhcg1 and Nhe3b, which have been shown to be involved in ammonium-dependent sodium uptake by zebrafish larvae acclimated to low-sodium water.

We provided direct experimental evidence for the extracellular cell surface localization of CA15a. This may deserve a comment since such localization has been supposed based on the presence of a signal peptide on the N terminus of its nascent precursor and the presence of a C-terminal GPI anchor signal (Udenfriend and Kodukula, 1995; Eisenhaber et al., 2000). In our transport metabolon model, therefore, CA15a is drawn to be anchored in the outer leaflet of the apical membrane of H-MRC *via* a GPI anchor. Such extracellular cell surface localization of CA IV (CA4) family members has been suggested only by indirect evidence such as the sensitivities to phosphatidylinositol-specific phospholipase C and membrane-impermeable inhibitors of CA (Waheed et al., 1992; Georgalis et al., 2006; Purkerson and Schwartz, 2007).

The incomplete inhibition of the Na^+^ uptake achieved by the knockdown with MOs may be due to incomplete depletion of the CA isoforms or the presence of an alternative pathway(s) for Na^+^ uptake, or both. Our result of Sodium Green is, however, apparently in contradiction with that of Lin et al. (2008) who showed a significantly higher influx of ^24^Na^+^ in *ca2a* and *ca15a* morphants and interpreted the ^24^Na^+^ result as due to upregulation of Nhe3b. The difference may be due to that Sodium Green measures the Na^+^ concentrations in MRCs while the ^24^Na^+^ assay estimates the whole body ^24^Na^+^ concentration accumulated not only through MRCs but also through other routes.

As mentioned above, through a systematic analysis combined with database mining, we established the members of the CA family that are responsible for the CA activity important for Na^+^ uptake in FW conditions and provided evidence for a concerted action of the CA family members (CA2a and CA15a) and a member of the sodium-proton exchanger family (Nhe3b) by forming a transport metabolon, in which transmembrane protein Nhe3b interacts with both CA2a and CA15a through its intracellular and extracellular regions, respectively. Furthermore as an extension of recent demonstrations of the presence of an ammonia transporter, Rhcg1, in zebrafish H-MRCs (Nakada et al., 2007) and its functional association with Nhe3 (Wu et al., 2010; Shih et al., 2012), we provided evidence for their close association (CA2a/15a-Nhe3b-Rhcg1) by the proximal ligation assay. Functional coupling of Rhcg1 and H^+^-ATPase has also been suggested (Nawata et al., 2007). A future direction of research would, therefore, be to determine if the CA2a/15a-Nhe3b-Rhcg1 complex exists as a component of a much higher-order transport metabolon including H^+^-ATPase, which couples Na^+^ uptake with excretion of H^+^ and ammonia, the end product of nitrogen metabolism in teleost fishes. To establish definitively the presence of such a complex, it would be necessary to determine the domains or amino acid sequences involved in the interaction as has already been established in the case of mammalian CA II-NHE1 (Li et al., 2002, 2006) and CA II-SLC26A6 (Alvarez et al., 2005).

## Conflict of Interest Statement

The authors declare that the research was conducted in the absence of any commercial or financial relationships that could be construed as a potential conflict of interest.

## Footnotes

^1^The following nomenclature was used here: tetrapod nucleotides, *ALL CAPITALS* (italicized); tetrapod proteins, ALL CAPITALS; non-tetrapod nucleotides, *all small cases* (italicized); non-tetrapod proteins, first letter capitalized and subsequent letters in small cases except CA to avoid confusion with calcium (Ca).
